# Favorable Alteration of Tumor Microenvironment by Immunomodulatory Cytokines for Efficient T-Cell Therapy in Solid Tumors

**DOI:** 10.1371/journal.pone.0131242

**Published:** 2015-06-24

**Authors:** Siri Tähtinen, Saija Kaikkonen, Maiju Merisalo-Soikkeli, Susanna Grönberg-Vähä-Koskela, Anna Kanerva, Suvi Parviainen, Markus Vähä-Koskela, Akseli Hemminki

**Affiliations:** 1 Cancer Gene Therapy Group, Department of Pathology and Transplantation Laboratory, Haartman Institute, University of Helsinki, Helsinki, Finland; 2 Department of Obstetrics and Gynecology, Helsinki University Central Hospital, Helsinki, Finland; 3 TILT Biotherapeutics Ltd, Helsinki, Finland; 4 Department of Oncology, Helsinki University Central Hospital, Helsinki, Finland; Mie University Graduate School of Medicine, JAPAN

## Abstract

Unfavorable ratios between the number and activation status of effector and suppressor immune cells infiltrating the tumor contribute to resistance of solid tumors to T-cell based therapies. Here, we studied the capacity of FDA and EMA approved recombinant cytokines to manipulate this balance in favor of efficient anti-tumor responses in B16.OVA melanoma bearing C57BL/6 mice. Intratumoral administration of IFN-α2, IFN-γ, TNF-α, and IL-2 significantly enhanced the anti-tumor effect of ovalbumin-specific CD8+ T-cell (OT-I) therapy, whereas GM-CSF increased tumor growth in association with an increase in immunosuppressive cell populations. None of the cytokines augmented tumor trafficking of OT-I cells significantly, but injections of IFN-α2, IFN-γ and IL-2 increased intratumoral cytokine secretion and recruitment of endogenous immune cells capable of stimulating T-cells, such as natural killer and maturated CD11c+ antigen-presenting cells. Moreover, IFN-α2 and IL-2 increased the levels of activated tumor-infiltrating CD8+ T-cells concomitant with reduction in the CD8+ T-cell expression of anergy markers CTLA-4 and PD-1. In conclusion, intratumoral administration of IFN-α2, IFN-γ and IL-2 can lead to immune sensitization of the established tumor, whereas GM-CSF may contribute to tumor-associated immunosuppression. The results described here provide rationale for including local administration of immunostimulatory cytokines into T-cell therapy regimens. One appealing embodiment of this would be vectored delivery which could be advantageous over direct injection of recombinant molecules with regard to efficacy, cost, persistence and convenience.

## Introduction

Adoptive T-cell therapies (ACT) are a potent approach for treating cancer. Immunotherapy using tumor-specific T-cells was first established by Steven Rosenberg in 1980’s and subsequently human trials of *ex vivo* expanded tumor-infiltrating lymphocytes (TILs) have shown promising results when combined to systemic high-dose interleukin-2 (IL-2) and lymphodepletion [1]. Importantly, significant toxicities and even mortality has been associated with these concomitant treatments, while TIL therapy *per se* has been considered safe [2,3]. More recently, approaches to genetically engineer peripheral blood T-cells have provided proof-of-concept data but modest response rates in advanced solid tumors [4,5]. In contrast, exceptional efficacy has been achieved in the treatment of CD19-expressing hematological malignancies using chimeric antigen receptor (CAR) T-cells [6,7], highlighting the inherent potential of the technology for any tumor type, including solid tumors, if critical obstacles such as T-cell hypofunction [8] can be overcome.

Several recombinant cytokines are routinely used in the treatment of cancer and other diseases [9]. Granulocyte macrophage—colony stimulating factor (GM-CSF) has been approved by FDA for the treatment of neutropenia due its capacity to stimulate the differentiation of bone marrow stem cells [10]. Interferon α2 (IFN-α2) is a type I interferon, which can activate different immune cells and has been utilized for decades in the treatment of melanoma and renal cell cancer [11]. Interferon γ (IFN-γ), a type II interferon, is FDA-approved for the therapy of granulomatous disease and severe osteopetrosis, and clinical studies for efficacy in oncological indications are ongoing [12]. Tumor necrosis factor α (TNF-α) is used in isolated limb perfusion of locally advanced melanoma or soft tissue sarcoma [9] due to its capacity to induce tumor cell apoptosis and subsequent immunological anti-tumor responses [13]. Lastly, interleukin-2 (IL-2) stimulates the growth, differentiation and survival of antigen-specific T-cells [14] and has been used as monotherapy for several different cancer types, including melanoma [15].

As all of the aforementioned cytokines have been shown to induce innate and/or adaptive immune responses against the established tumor both in preclinical and clinical settings [9,16], we hypothesized that local administration of recombinant cytokines could manipulate the tumor microenvironment in favor of adoptive T-cell therapy. Confirming our initial hypothesis, more than one of the studied five cytokines proved to be able to modulate the microenvironment and reduce the tumor resistance to cytotoxic CD8+ T-cells. These preclinical results support the use of intratumorally administrated, carefully selected cytokines in combination with adoptive T-cell therapy.

## Materials and Methods

### Cells and recombinant murine cytokines

Murine melanoma B16 cells expressing ovalbumin (OVA) [17,18] were a generous gift from Prof. Richard Vile (Mayo Clinic, MN, September 30^th^ 2010). B16.OVA were maintained in RPMI 1640, 10% FBS, 5 mg/ml G418, 20 mM L-Glutamine, 1x Pen/Strep solution and cultured at 37°C and 5% CO_2_. Carrier-free murine cytokines interferon α2, interferon γ (from eBioscience, San Diego, CA), IL-2 and GM-CSF (from Invitrogen, Waltham, MA) were thawed after receipt, reconstituted in PBS at 100 μg/ml and aliquots stored at -80°C until use.

### Isolation and expansion of T-cells

C57BL/6-Tg(TcraTcrb)1100Mjb/J (OT-I) mice are widely used models in immunology and these mice have transgenic T-cell receptors designed to recognize OVA residues 257–264 (SIINFEKL) in the context of H-2Kb. Spleen and lymph nodes were collected from OT-I mice, processed into single-cell suspension and treated with ACK lysing buffer to remove red blood cells. CD8a+ T-cells were enriched by depleting non-target cells with mouse CD8 (Ly-2) Microbeads (Miltenyi Biotech, Bergisch Gladbach, Germany). Enriched T-cells were expanded for 7 days in RPMI 1640 supplemented with 10% FBS, 20 mM L-Glutamine, 1x Pen/Strep solution, 15 mM HEPES, 50 μM 2-mercaptoethanol, 1 mM Na pyruvate, 160 ng/ml recombinant murine IL-2 (R&D Systems, Minneapolis, MN) and 300 ng/ml soluble anti-mouse CD3e antibody (clone 145-2C11, Abcam, Cambridge, UK). Last polyclonal activation with IL-2 and anti-mouse CD3 was peformed 3 days prior to adoptive transfer.

### Ethics Statement

This study was carried out in strict accordance with the recommendations in the Act on the Protection of Animals Used for Scientific or Educational Purpose (497/2013) and Government Decree on the Protection of Animals Used for Scientific or Educational Purposes (564/2013). The protocols were approved by the National Animal Experiment Board of the Regional State Administrative Agency of Southern Finland (permit number: ESAVI/4621/04.10.03/2012). All injections were performed under isoflurane anesthesia and all efforts were made to minimize suffering.

### Animal experiments

4–7-week-old C57BL/6 immunocompetent female mice were implanted subcutaneously with 2.5 x 10^5^ B16.OVA cells in 50 μl RPMI, 0% FBS, in the right flank. Ten days post tumor implantation, mice were divided into groups and tumors (~3 mm minimum diameter) were left non-injected or injected with either 50 μl phosphate buffered saline (PBS) or carrier-free recombinant murine cytokines in 50 μl PBS (Table 1). Mice received 10 doses of recombinant cytokines intratumorally in total (S1 Fig). On the first day of the intratumoral treatment, the mice were also adoptively transferred with 2 x 10^6^ CD8a-enriched and expanded splenocytes from OT-I mice. The OT-I cells were administered into intraperitoneal cavity in 100 μl RPMI, 0% FBS, as it has been shown that intraperitoneal injections of OT-I mimic the kinetics of intravenous delivery [19]. Tumor growth of mice was monitored every 2–3 days by using electronic calipers and volume was calculated as 0.52 x length x width^2^. Mice were examined every day and euthanized before the designated experimental endpoint of day 14 when the tumor became ulcerated or when one of two diameters reached 18 mm.

**Table 1:** Doses of recombinant cytokines.

Group	Dose/Mouse/Day (μg)	Dose/Mouse/Day (U)
Non-injected	-	-
PBS	-	-
GM-CSF	1	N/A*
IFN-α2	0,3	3 000
IFN-γ	1,75	10 000
TNF-α	0,5	N/A*
IL-2	0,3	3 000

*N/A = not assessed

### Tissue processing for Flex Set analysis

Mice were euthanized and 10–100 mg of tumor tissue was frozen in 2 ml microcentrifuge tubes on dry ice and stored at -80°C. Prior to processing ice-cold PBS supplemented with 0.1% BSA and protease inhibitor cocktail (Sigma-Aldrich, St. Louis, MO) was added and the tumor pieces were homogenized by Tissue Master 125 rotor (Omni International, Kennesaw, GA). Tumor homogenate was spun at 2000 RCF 10 min +4°C and the supernatant was analyzed with CBA Flex Set cytokine beads (BD, Franklin Lakes, NJ) on BD Accuri C6 flow cytometer with FCAP Array software (BD) per manufacturer’s instructions.

### Tissue processing for flow cytometry

Mice were euthanized and tumors were processed for flow cytometric analysis by pushing the tumor tissue through a 70 μm sterile strainer using a 1 ml syringe plunger. RPMI 1640 supplemented with 10% FBS, 20 mM L-Glutamine, 1x Pen/Strep was added and the single-cell solution was cultured at 37°C and 5% CO_2_ for 24 hours, after which cells were either analyzed directly by flow cytometry or frozen at -80°C for later analysis.

### Flow cytometry

Tumor cell samples were stained according to manufacturer instructions with respective commercial antibodies validated by the supplier (Table 2). The labeled cells were centrifuged at 500 RCF for 5 min and the pellet was resuspended in Flow Cytometry Staining Buffer (eBioscience). For T-cell activation assay tumor samples were treated with intracellular protein transport inhibitor brefeldin A (eBioscience) and Cell Stimulation Cocktail containing PMA and ionomycin (eBioscience) at 37°C and 5% CO_2_ for 6 hours. After stimulation the cells were stained for surface markers, fixed and permeabilized prior to intracellular staining. All cell samples were analyzed on BD Accuri C6 flow cytometer with CFlow Sampler software (BD) counting at least 100000 events per sample.

**Table 2:** List of antibodies used.

Antibody	Monoclonal or polyclonal	Host species	Commercial supplier	Catalogue number	Concentration (per sample)
CD8b-FITC	monoclonal	rat	eBioscience	11-0083-85	0,5 μg
Foxp3-APC	monoclonal	rat	eBioscience	17-5773-82	1 μg
CD25-PE	monoclonal	rat	eBioscience	12-0251-82	0,125 μg
CD19-PE	monoclonal	rat	eBioscience	12-0193-82	0,125 μg
H-2Kb-PE	monoclonal	mouse	eBioscience	12-5958-82	0,25 μg
H-2Kb-SIINFEKL-PeCy7	monoclonal	mouse	eBioscience	25-5743-80	0,125 μg
CD8a-APC	monoclonal	rat	eBioscience	17-0081-82	0,125 μg
CD45-APC	monoclonal	rat	eBioscience	17-0451-82	0,125 μg
CTLA-4-PE	monoclonal	armenian hamster	eBioscience	12-1522-81	0,25 μg
PD-1-PeCy7	monoclonal	armenian hamster	eBioscience	25-9985-80	1 μg
NK1.1-FITC	monoclonal	mouse	eBioscience	11-5941-81	0,5 μg
F4/80-APC	monoclonal	rat	eBioscience	17-4801-82	0,5 μg
CD44-FITC	monoclonal	rat	eBioscience	11-0441-81	0,5 μg
CD62L-PE	monoclonal	rat	eBioscience	12-0621-81	0,125 μg
CD69-PeCy7	monoclonal	armenian hamster	eBioscience	25-0691-82	0,5 μg
IFN-γ-APC	monoclonal	rat	eBioscience	17-7311-82	0,125 μg
CD4-PerCP.Cy5.5	monoclonal	rat	BD	550954	0,4 μg
CD3-PeCy7	monoclonal	rat	BD	560591	0,2 μg
CD11c-FITC	monoclonal	armenian hamster	BD	553801	0,5 μg
Gr-1-FITC	monoclonal	rat	BD	553127	0,5 μg
Ly6G-PE	monoclonal	rat	BD	551461	0,3 μg
CD11b-PerCP-Cy5.5	monoclonal	rat	BD	550993	0,3 μg
Ly6C-APC	monoclonal	rat	BD	560595	0,3 μg
CD86-PE	monoclonal	rat	BD	553692	0,3 μg
CD3-APC	monoclonal	armenian hamster	BD	553066	0,3 μg
CCR7-PerCP-Cy5.5	monoclonal	rat	BD	560812	0,5 μg
CD206-FITC	monoclonal	rat	Biolegend	141704	0,125 μg
SIINFEKL-pentamer-APC	ND*	ND*	Proimmune	F093-4B	10 μl

*ND = not determined

### Statistical analysis

Statistics was performed with GraphPad Prism 6 (GraphPad Software Inc., San Diego, CA) and SPSS version 21 (SPSS IBM, New York, NY). One-way ANOVA followed by Tukey’s post-hoc test was used for comparison of multiple groups. Log-transformed tumor volumes were analyzed by repeated measures ANOVA. Differences were considered statistically significant when P values were < 0.05.

## Results

### Anti-tumor efficacy is achieved by intratumoral administration of IFN-α2, IFN-γ, TNF-α and IL-2 but not GM-CSF

To study the impact of local immunomodulatory treatment on tumor microenvironment following adoptive T-cell therapy, we chose the well-established standard-of-the-field syngeneic melanoma model B16 expressing chicken ovalbumin (OVA) as a model tumor-associated antigen. This model is highly immunosuppressive and thus resembles many advanced human melanomas representing the population in need of experimental therapies. Mice bearing B16.OVA tumors were treated with intraperitoneal administration of 2x10^6^ OVA peptide SIINFEKL-specific, CD8a+ enriched OT-I cells while the tumors were either not injected or injected intratumorally with phosphate buffered saline (PBS), GM-CSF, IFN-α2, IFN-γ, TNF-α or IL-2. These intratumoral treatments were continued for five days per week for a total of 2 weeks (S1 Fig).

**Fig 1:**
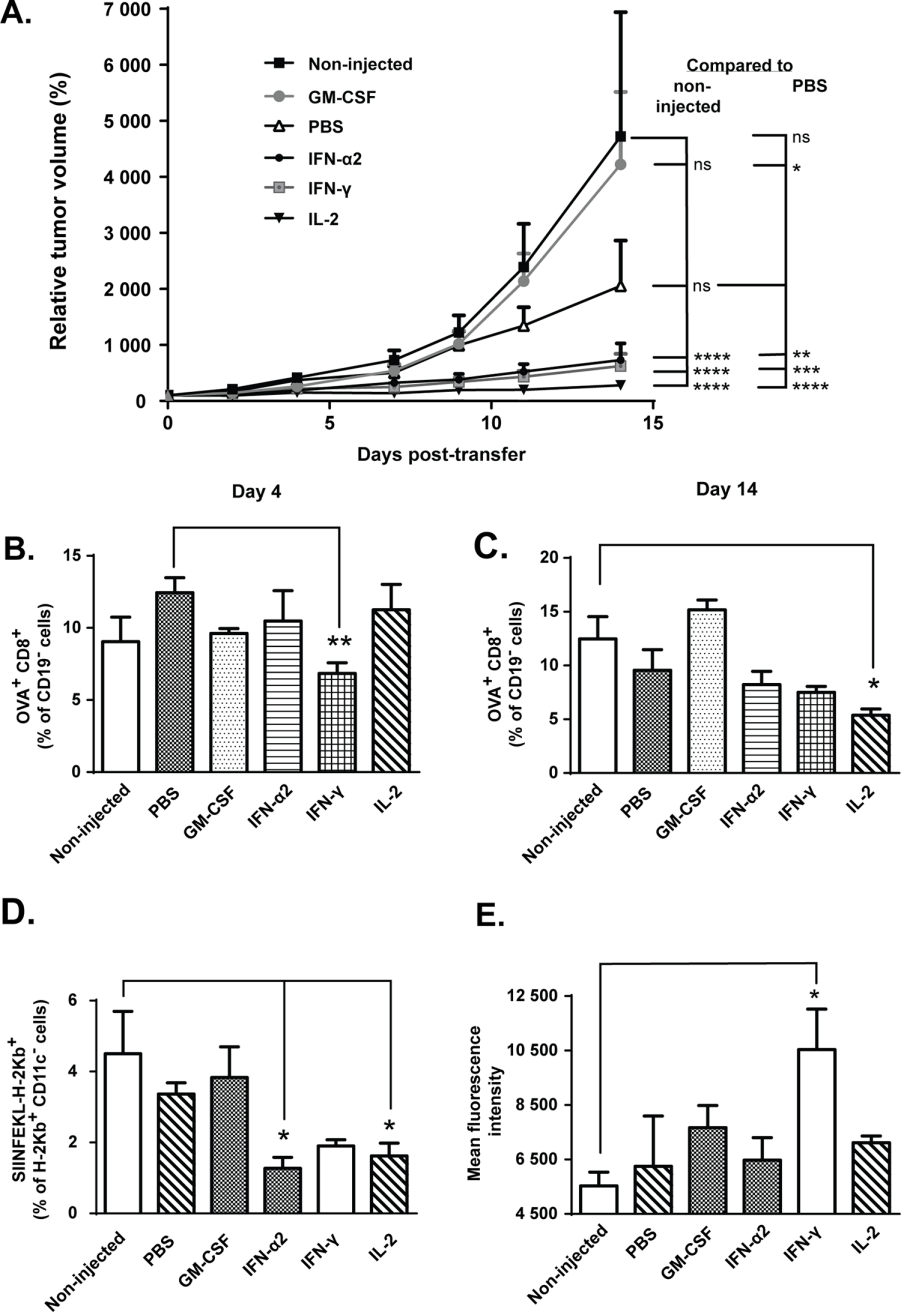
IFN-α2, IFN-γ and IL-2 augment anti-tumor efficacy but do not increase tumor-accumulation of transferred cells. Mice bearing syngeneic B16.OVA tumors were adoptively transferred with 2x10^6^ CD8a^+^ enriched, polyclonally activated OT-I lymphocytes intraperitoneally and tumors were either left non-injected or injected with PBS or recombinant cytokine in PBS (n = 10). (a) Tumor growth was monitored every 2–3 days with an electronic caliper. Due to variation in tumor sizes at the beginning of the experiment, the results are represented as relative change compared to day 0 volume, which was set at 100%. (b-c) Levels of OT-I cells in tumors were quantified on days 4 (b) and 14 (c) post-transfer by pentamer staining and flow cytometry. (d) Proportion of major histocompatibility complex (MHC) class I molecules presenting OVA-derived peptide SIINFEKL and (e) mean fluorescence intensity (MFI) of mouse MHC class I H-2kb from tumor samples was assessed by flow cytometry on day 14 post-transfer (n = 5). Data presented as mean ± SEM. *P≤ 0.05, **P≤ 0.01, ***P≤ 0.001, ****P≤ 0.0001 by repeated measures ANOVA (a) or one-way ANOVA followed by Tukey’s post-hoc test (b-e).

In accordance with typical clinical outcomes in melanoma T-cell therapy in the absence of preconditioning [20], cells alone resulted in poor growth control of established tumors (Fig 1A; non-injected and PBS-injected groups). Instead, administration of intratumoral IFN-α2, IFN-γ and IL-2 resulted in superior treatment efficacy over control groups (Fig 1A and Fig A in S2 Fig). In addition, TNF-α was found effective in curing 80% of mice by day 14 post-transfer (Fig B in S2 Fig). As an interesting side note, daily injections of phosphate buffered saline also added to the anti-tumor effect of OT-I T-cell therapy, supporting previous notions that any damage to the tumor can result in immune response [21,22]. While many of the cytokines injected improved the anti-tumor effect of OT-I therapy, GM-CSF resulted in growth-stimulatory effect when compared to PBS injected control tumors (Fig 1A), as described earlier by Obermueller et al [23].

### Levels of ovalbumin-specific CD8+ T-cells do not correlate with treatment efficacy

To assess the degree of tumor-antigen specific T-cell infiltration in tumors as a possible explanation for the observed anti-tumor effects with the cytokine combinations, total CD8+ T-cells specific for MHC-I-loaded chicken ovalbumin SIINFEKL peptide (endogenous + transferred OT-I cells) were quantified by flow cytometry on days 4 and 14 post-transfer. Interestingly, the levels of tumor-infiltrating pentamer-positive CD8+ T-cells were lower in IFN-γ–treated mice compared to PBS-injected controls on day 4 post-transfer and in IL-2-injected mice compared to non-injected controls on day 14 post-transfer (Fig 1B–1C). At the same time, however, the level of putative non-DC target (tumor) cells, identified as CD11c^-^ cells presenting the OVA-derived peptide SIINFEKL in context of MHC class I, was significantly lower in IFN-α2 and IL-2-injected tumors compared to non-injected controls on day 14 post-transfer (Fig 1D). It is possible that any target cells presenting OVA-peptides on their H-2Kb molecules, including melanoma cells, were efficiently killed by tumor-reactive cytotoxic T-cells earlier on, thus leading to antigen-loss variants in the tumor cell population through immunoediting [24]. In order to gain more insight into CD8+ T-cell-mediated tumor control, we assessed overall expression of MHC class I, depicted as H-2Kb^+^ cells by flow cytometry on day 14 post-transfer. MHC-I expression was enhanced by intratumoral IFN-γ but not by any other cytokines (Fig 1E). Thus, MHC-I expression in the tumors did not directly predict anti-tumor efficacy with the cytokine combinations.

### Combination of local cytokine injections and adoptive T-cell transfer is associated with changes in both pro- and anti-inflammatory cytokine levels in tumors

In order to gain further insight into the possible anti-tumor mechanisms of adoptive CD8+ T-cell transfer combined with intratumoral cytokine injections, which seemingly did not involve increased trafficking of CD8+ T-cells or increased intratumoral MHC-I expression (Fig 1B–1E), we analyzed tumors at the study endpoint for several central immunomodulatory cytokines: T-cell growth factor IL-2, pro-inflammatory cytokines IFN-γ, TNF-α and heterodimeric IL-12p70, all of which are associated with T-cell activation and Th1 polarization; anti-inflammatory cytokine IL-10, secreted by regulatory T-cells and Th2 cells; innate monocyte/NK immune activators GM-CSF and IL-1β; as well as the “acute phase” cytokine IL-6 [25,26].

**Fig 2:**
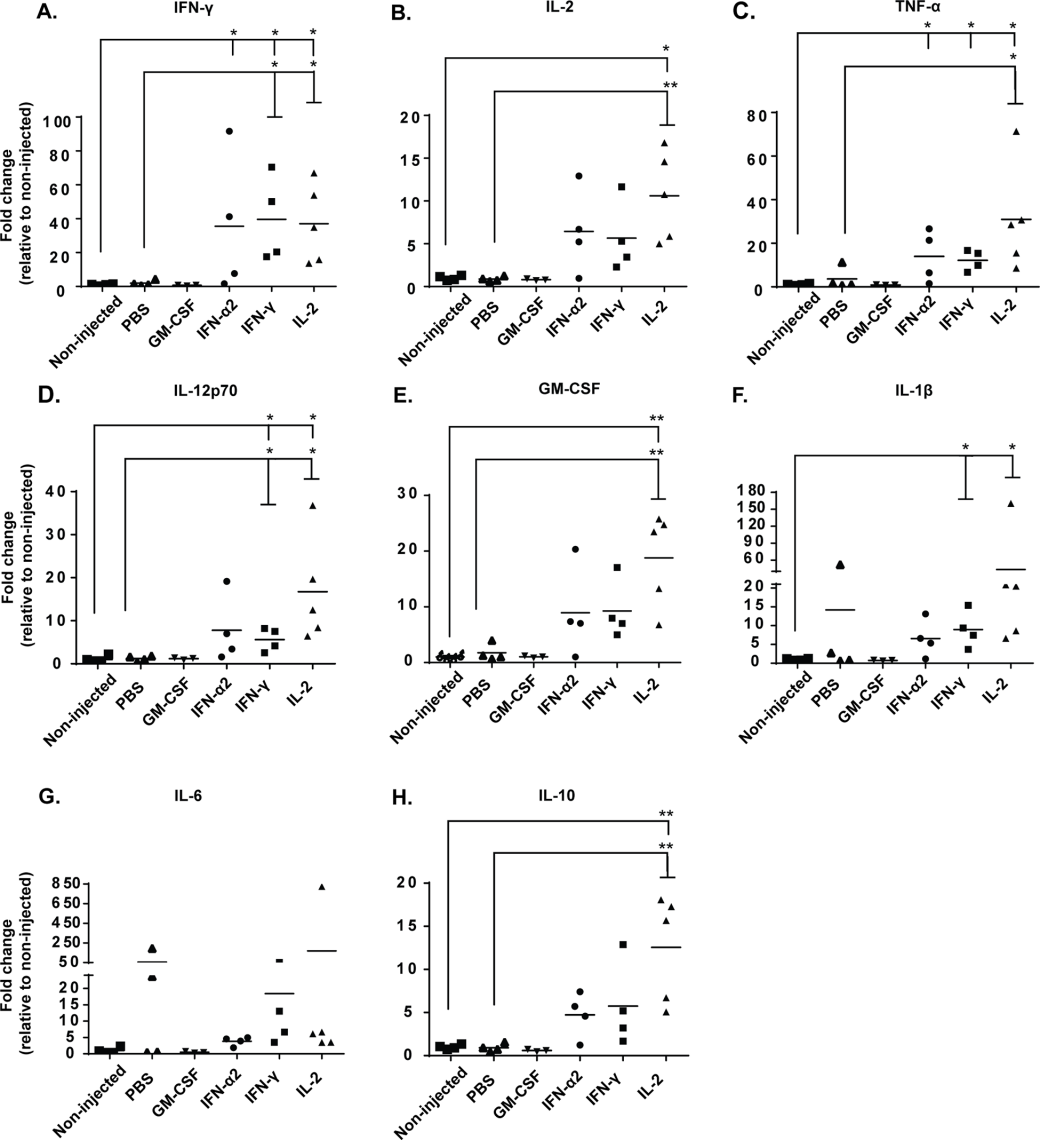
Recombinant cytokines induce intratumoral, endogenous secretion of cytokines associated with immune cell activation. B16.OVA-bearing mice were treated intraperitoneally with 2x10^6^ CD8a^+^ enriched OT-I lymphocytes and treated intratumorally with either PBS or recombinant cytokine (in PBS) or left non-injected. Levels of (a) IFN-γ, (b) IL-2, (c) TNF-α, (d) IL-12p70, (e) GM-CSF, (f) IL-1β, (g) IL-6 and (h) IL-10 from tumor homogenates were measured with CBA Flex sets on day 14 post-transfer (n = 3–5). Horizontal lines represent median values. *P≤ 0.05 and **P≤ 0.01 byone-way ANOVA followed by Tukey’s post-hoc test.

Remarkably, intratumoral injection with IFN-γ and IL2 increased the secretion of all of these cytokines compared to non-injected and PBS-injected tumors, with no clear delineation toward either pro-inflammatory (Fig 2A–2D) or anti-inflammatory profiles (Fig 2E–2H). While it was not possible to separate the relative levels of the injected recombinant cytokines from the endogenously induced cytokines, the levels of the other analyzed cytokines were still increased, confirming that local cytokine treatment modulates the overall cytokine balance in the tumor. This secretion of cytokines in the tumor may be an indicator of heightened immune detection and destruction of the established tumor [27–29], as the control groups show very low levels of endogenous cytokines and subsequently poor tumor growth control. Interestingly, daily injections of GM-CSF did not result in measurable increase of total GM-CSF over background in the tumors at the sampled endpoint (Fig 2E), implying rapid turnover of the cytokine.

### Local administration of IL-2 decreases the total number of CD4+ TILs but induces CD4+ T-cell polarization into Tregs

Since we saw no clear skewing of the intratumoral cytokine balance towards tumor rejection, we proceeded by analyzing the phenotypes of immune cells present in the tumors. On day 14 post-transfer IFN-γ- and IL-2-treated tumors contained more CD45^+^ leukocytes than control tumors (Fig A in S3 Fig), whereas the total amount of CD3^+^ T-lymphocytes did not significantly differ between the control and treatment groups (Fig B in S3 Fig). Local administration of IL-2 decreased the levels of tumor-infiltrating CD4^+^ T-cells compared to non-injected control mice (Fig C in S3 Fig). In addition, PBS and IL-2 injections resulted in CD4^+^ T-cell polarization toward regulatory phenotype (Foxp3^+^ CD25^+^ CD4^+^) within the studied T-cell population (Fig D in S3 Fig), which for IL-2 was expected [30].

### Intratumoral IFNa2 and IL-2 increase the accumulation of immune cells capable of stimulating CD8+ T-cells

**Fig 3:**
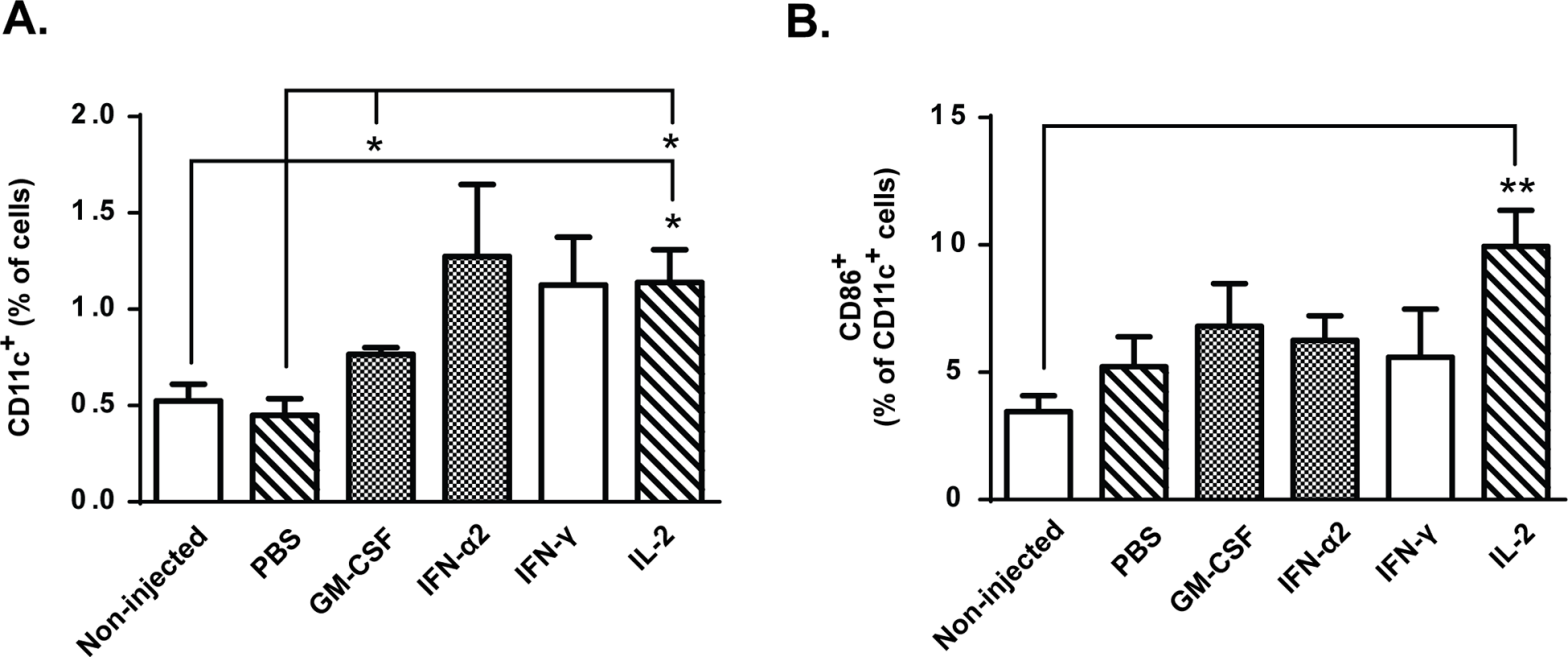
Intra-tumor accumulation of antigen-presenting cells (APCs) is increased by GM-CSF and IL-2. B16.OVA bearing mice were treated with adoptive transfer of 2x10^6^ CD8a^+^ enriched OT-I lymphocytes intraperitoneally and tumors were either injected with PBS or recombinant cytokine in PBS or left non-injected (n = 5). (a) Levels of CD11c^+^ dendritic cells and (b) proportion of dendritic cells expressing maturation marker CD86 on cell surface were analyzed on day 14 post-transfer from tumors. Data presented as mean ± SEM. *P ≤ 0.05 and **P≤ 0.01 by one-way ANOVA followed by Tukey’s post-hoc test.

**Fig 4:**
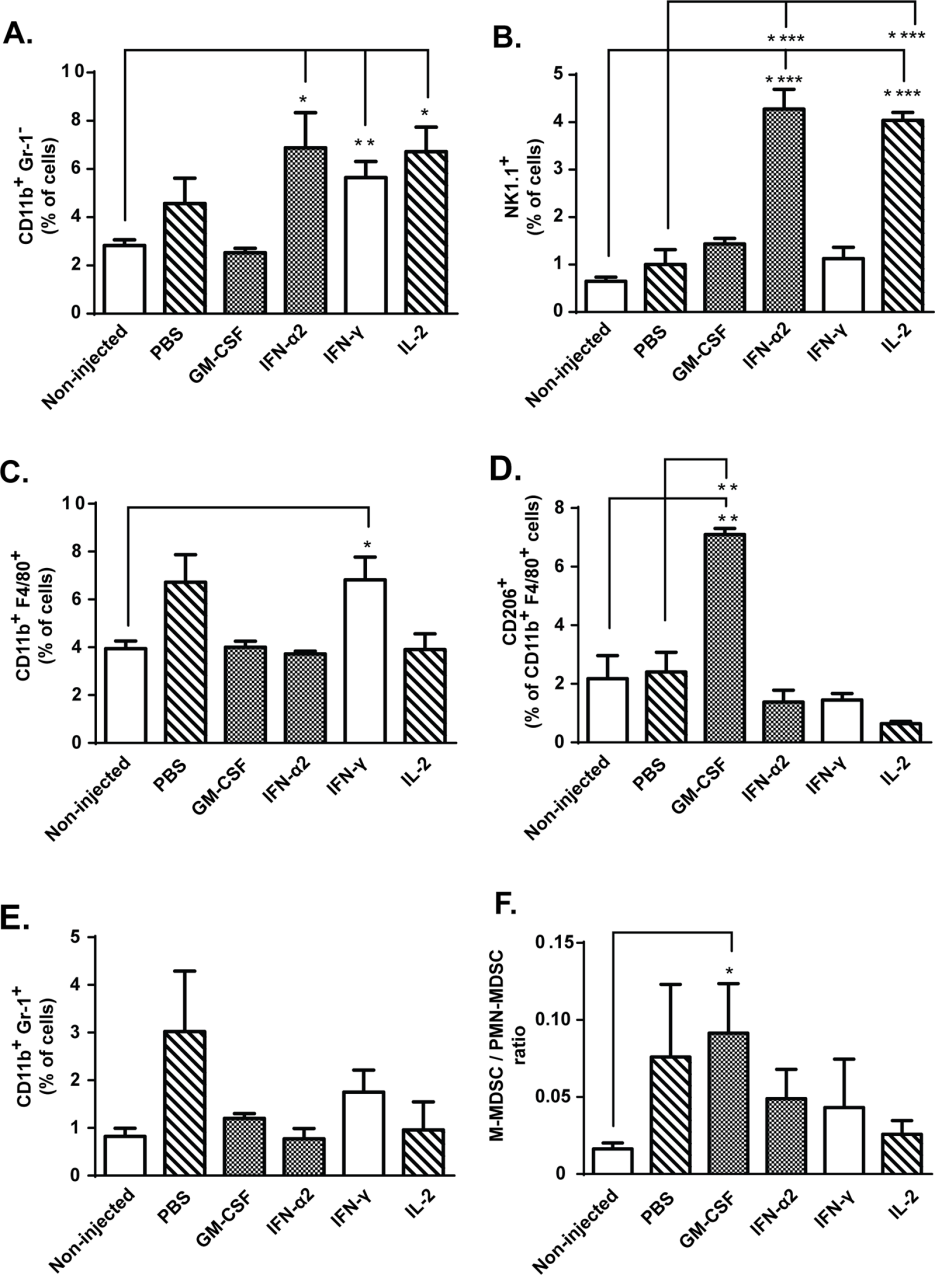
Intratumoral myeloid cell subsets are influenced by local cytokine therapy. Mice bearing subcutaneous B16.OVA tumors received intraperitoneal transfer of 2x10^6^ CD8a^+^ enriched OT-I lymphocytes and intratumoral injections of either PBS or recombinant cytokine in PBS (n = 5). Levels of tumor-infiltrating (a) CD11b^+^ myeloid cells, (b) NK1.1^+^ natural killer cells, (c) CD11b^+^ F4/80+ macrophages, (d) suppressive M2 macrophages (characterized by surface expression of CD206), (e) CD11b^+^ Gr-1^+^ myeloid-derived suppressor cells (MDSC) and (f) ratio of monocytic (M) to polymorphonuclear (PMN) MDSCs were assessed from tumors on day 14 post-transfer by flow cytometry. Data presented as mean ± SEM. *P ≤ 0.05, **P≤ 0.01, ***P≤ 0.001 and ****P≤ 0.0001 byone-way ANOVA followed by Tukey’s post-hoc test.

T-cell activity is regulated by antigen-presenting cells (APCs), which can either promote activation of tumor-specific T-cells or induce antigen-specific peripheral tolerance in absence of co-stimulatory signals such as CD86 [31]. Following intratumoral immunomodulation with GM-CSF and IL-2, the total levels of dendritic cells were increased over control groups (Fig 3A). Analysis of the maturation status of these cells revealed that only *in situ* administration of IL-2 resulted in higher proportion of intratumoral CD11c^+^ CD86^+^ DCs compared to non-injected control group (Fig 3B). In addition, the number of tumor-infiltrating natural killer (NK) cells was increased in IFN-α2 and IL-2 treated mice compared to non-injected and PBS controls (Fig 4B). Thus, the greater proportion of mature DCs and NK cells following IL-2 administration compared to GM-CSF administration could have contributed to the superior anti-tumor effects of IL-2.

### In situ administration of recombinant GM-CSF results in tumor-infiltrating myeloid cells polarization into immunosuppressive phenotype

In order to assess influence of the cytokine treatments on tumor composition as a possible explanation for the observed anti-tumor effects, we characterized the myeloid cell populations in the tumors. Total number of tumor-infiltrating CD11b^+^ Gr-1^-^ myeloid cells was increased in IFN-α2, IFN-γ and IL-2 treated groups compared to non-injected control mice (Fig 4A). As NK-cells and F4/80+ macrophages account for most of the CD11b^+^ cells in these treatment groups (Fig 4B–4C), it is possible that the quality rather than quantity of tumor-infiltrating myeloid cells determines if tumors are rendered sensitive to killing by cytotoxic T-cells. Further analysis of macrophage polarization revealed that intratumoral GM-CSF injection skewed tumor-infiltrating macrophages towards immunosuppressive M2 phenotype, characterized by CD206 expression (Fig 4D). By contrast, as IFN-γ-treated tumors contained high levels of endogenous cytokines TNF-α, IL-12p70 and IL-1β (Fig 2C,2D, 2F and 2G) and the tumor-infiltrating macrophages did not express putative M2 marker CD206 (Fig 4D), we find it likely that IFN-γ instead skewed the macrophages towards M1 phenotype [32].

In addition to tumor-associated macrophages (TAM), intratumoral administration of exogenous GM-CSF also resulted in increased ratio of monocytic (M-MDSC, CD11b^+^Gr1^+^Ly6G^-^Ly6C^high^) over polymorphonuclear (PMN-MDSC, CD11b^+^Gr1^+^Ly6G^+^Ly6C^low^) myeloid-derived suppressor cells (Fig 4E–4F). Although both are part of the immune population suppressing T-cell functions, in some cases M-MDSCs have been considered more immunosuppressive than PMN-MDSCs [33].

### Immunomodulation through IFN-α2, IFN-γ and IL-2 results in changes in tumor-infiltrating T-cell phenotypes

**Fig 5:**
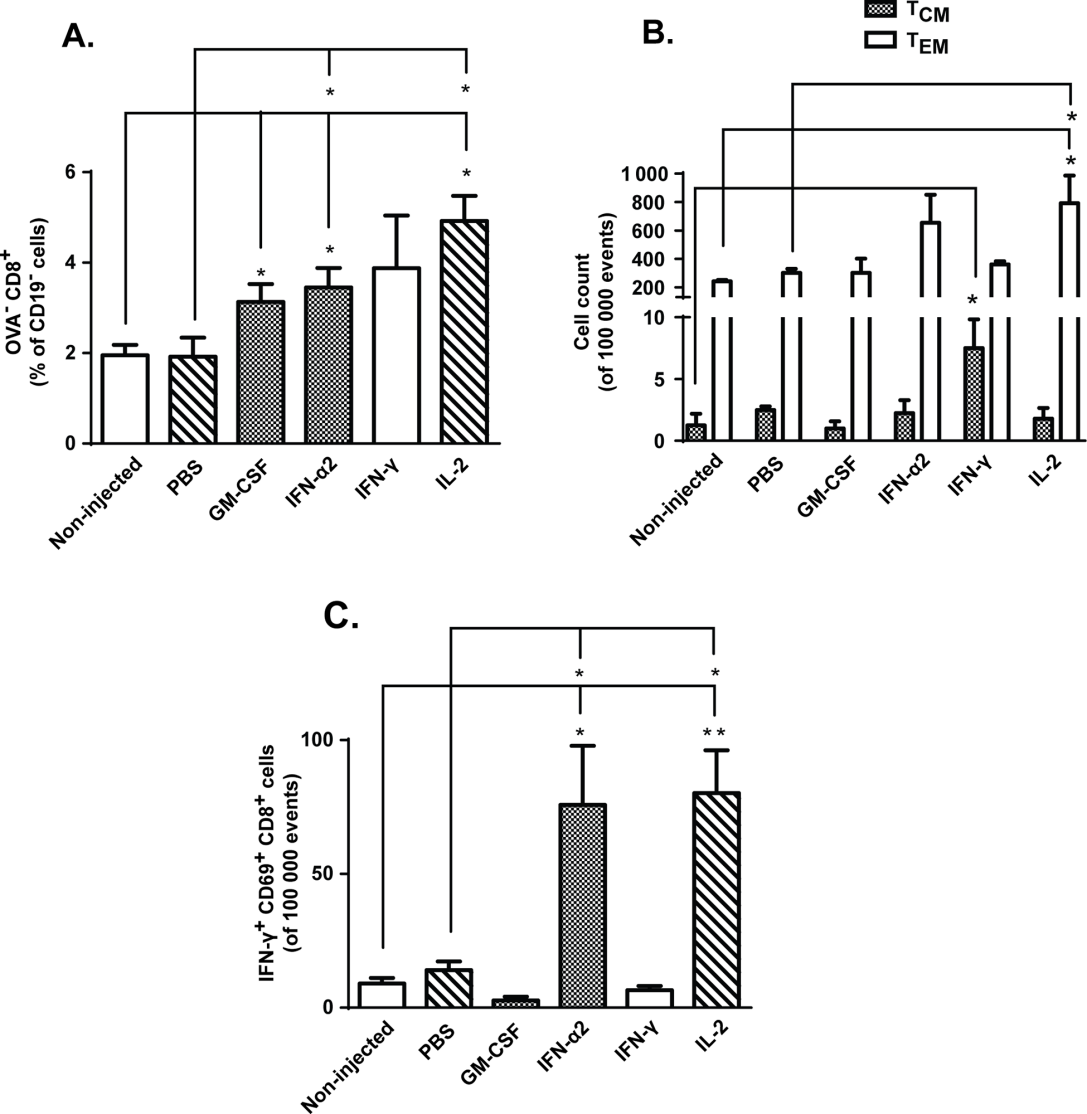
IFN-α2, IFN-γ and IL-2 treatment leads to changes in CD8+ TIL phenotypes. Mice harboring subcutaneous B16.OVA tumors were treated intraperitoneally with 2x10^6^ CD8a^+^ enriched OT-I lymphocytes and injected intratumorally with either PBS or recombinant cytokine in PBS or left non-injected (n = 5). (a) Levels of tumor-infiltrating endogenous (non-OVA) CD8^+^ T-cells and (b) count of central memory (T_CM_) and effector memory (T_EM_) T-cells were assessed from tumors on day 14 post-transfer by flow cytometry. (c) Activation status of tumor-infiltrating CD8+ T-cells was evaluated on day 14 by expression of CD69 and IFN-γ following PMA/Ionomycin stimulation *ex vivo*. Data presented as mean ± SEM. *P ≤ 0.05 and **P≤ 0.01 by one-way ANOVA followed by Tukey’s post-hoc test.

As we did not see evidence of increased tumor-accumulation of transferred OVA-specific OT-I cells following cytokine treatments, we decided to analyze the phenotype and activation status of tumor-infiltrating CD8+ T-cells. Interestingly, tumors treated with GM-CSF, IFN-α2 and IL-2 contained more endogenous, OVA^-^ CD8^+^ T-cells than non-injected tumors (Fig 5A). Further analysis revealed that some of these CD8^+^ TILs were targeting endogenous melanoma-associated antigens TRP-2 and gp100 (Figs E and F in S3 Fig), suggesting repertoire expansion following adoptive T-cell transfer [34,35]. In addition, local immunomodulation with IFN-γ resulted in increased levels of CD44^high^CD62L^high^CCR7^high^ central memory T-cells (T_CM_), whereas intratumoral IL-2 treatment led to increase in CD44^high^CD62L^low^CCR7^low^ effector memory T-cells (T_EM_) (Fig 5B). As previously reported [36], IL-2 promotes T-cell differentiation to T_EM_ cells, which have reduced proliferative capacity but can produce effector cytokines such as IFN-γ (depicted in Fig 2A). More importantly, PMA/Ionomycin stimulation of tumor suspensions revealed that IFN-α2 and IL-2 –treated tumors contained higher number of IFN-γ^+^ CD69^+^ CD8^+^ TILs compared to either control groups (Fig 5C), suggesting either increased tumor-infiltration of activated CD8+ T-cells or *in situ* activation of TILs following cytokine treatment.

### Downregulation of anergy markers on CD8+ TILs is achieved following IFN-α2, IFN-γ and IL-2 treatment

**Fig 6:**
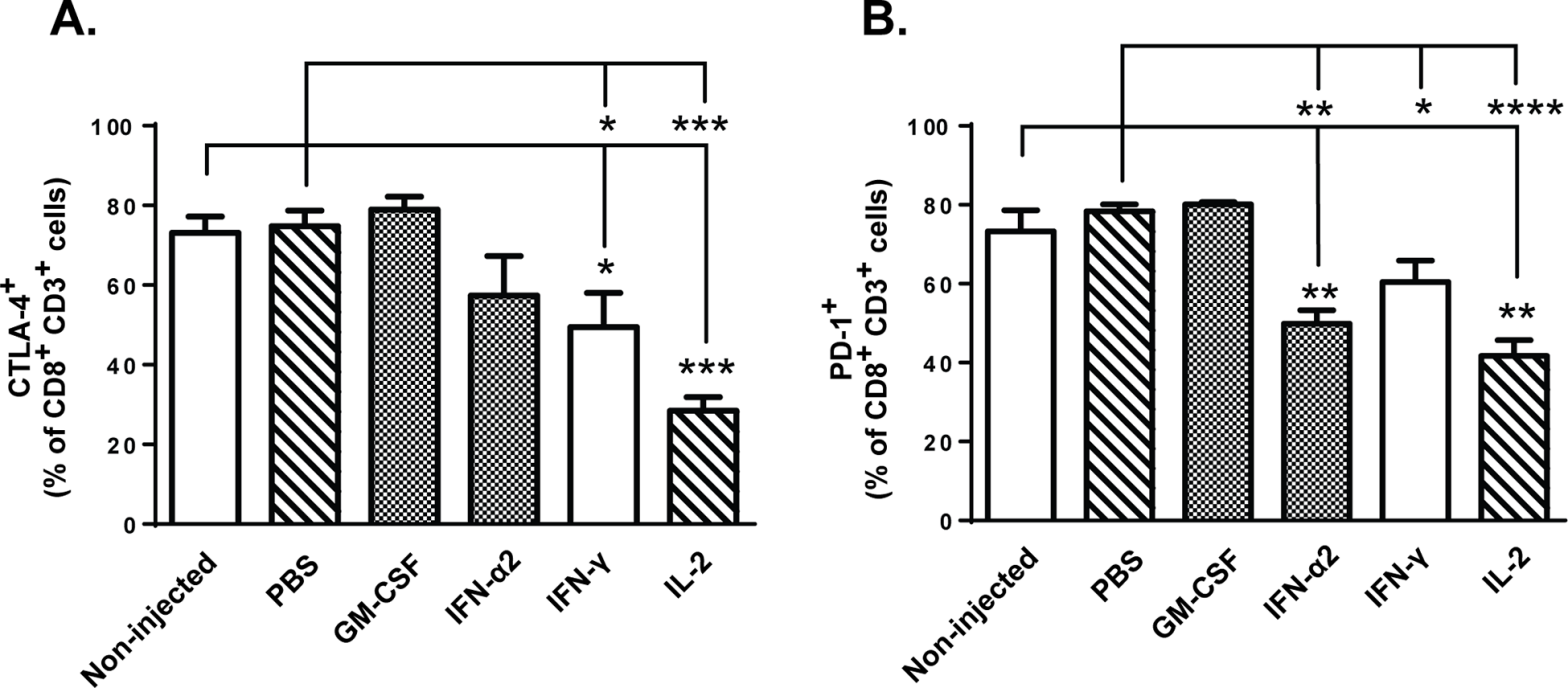
Expression of anergy markers on CD8+ TILs are downregulated following IFN-α2, IFN-γ and IL-2 treatments. Mice bearing subcutaneous B16.OVA tumors were injected with 2x10^6^ CD8a^+^ enriched OT-I lymphocytes into peritoneal cavity and beginning on the same day, tumors were injected with either PBS or recombinant cytokine in PBS or left non-injected (n = 5). Proportion of CD3^+^ CD8^+^ TILs expressing surface anergy markers (a) CTLA-4 and (b) PD-1 was analyzed by flow cytometry on day 14 post-transfer. Data presented as mean ± SEM. *P ≤ 0.05, **P≤ 0.01, ***P≤ 0.001, ****P≤ 0.0001 by one-way ANOVA followed by Tukey’s post-hoc test.

As TIL hypofunction has been associated with upregulation of surface inhibitory receptors [8], we wanted to study whether observed changes in tumor microenvironment also affect the expression of anergy/exhaustion markers CTLA-4 and PD-1 on CD8+ T-cells. Flow cytometric analysis of tumor-infiltrating lymphocytes revealed that administration of IFN-γ and IL-2 downregulated the expression of CTLA-4 on CD3+ CD8+ TILs compared to control groups on day 14 post-transfer (Fig 6A). Furthermore, significant reduction in expression of PD-1 on these tumor-infiltrating T-cells was observed following IFN-α2, IFN-γ and IL-2 treatment on day 14 (Fig 6B). These effects seemed to develop over time, since only IL-2 could downregulate CTLA-4 expression already on day 4 post-transfer (Fig A in S4 Fig) and IFN-γ treated tumors contained high levels of PD-1+ TILs at the early time point (Fig B in S4 Fig). Notably, the high expression of both CTLA-4 and PD-1 on CD8+ T-cells infiltrating the GM-CSF—treated tumors on both time points suggested that the tumor microenvironment remained highly immunosuppressive following cytokine treatment and thus implicates that administration of recombinant GM-CSF intratumorally may not be optimal for T-cell function. Taken together, our results indicate that local tumor treatment with carefully selected immunomodulatory cytokine (such as IFN-α2, IFN-γ and IL-2) can result in favorable alteration of tumor microenvironment and thus affect T-cell activity within the tumor.

## Discussion

Although immunotherapies based on gene-engineered T-cells have shown impressive clinical success in the treatment of hematological cancers such as chronic lymphocytic leukemia (CLL) and acute lymphoblastic leukemia (ALL) [6,7], the application into solid tumor types has remained difficult due to several obstacles including functional impairment of T-cell function following infiltration into tumor [4,5,8]. This T-cell hypofunction is induced by the strongly immunosuppressive tumor microenvironment, which is characterized by the lack of immune cells capable of activating anti-tumor effector cells and/or by the excess of immunosuppressive cell populations. In accord with clinical observations, our experiments showed that ACT-mediated anti-tumor immune responses, in the absence of preconditioning, are not potent enough to control tumor growth even in mice, as B16.OVA tumor-bearing mice treated with T-cells only displayed poor growth inhibition (Fig 1A). It has been previously suggested that resistance of solid tumors to adoptive T-cell therapy is the result of an imbalance between the number and/or activation status of tumor-infiltrating effector and suppressor immune cells [4]. Our aim was to manipulate this balance in favor of anti-tumor responses by using immunomodulatory cytokines administered intratumorally. Notably, injections of IFN-α2, IFN-γ, TNF-α and IL-2 markedly improved the anti-tumor effect of ACT, while treatment with GM-CSF resulted in stimulation of tumor growth (Fig 1A, S2 Fig). In addition, differences in efficacy and immune cell composition of non-injected and PBS-injected control tumors also revealed that mere physical manipulation of the tumor by a needle can affect the tumor microenvironment and cause inflammation that results in minor (but not statistically significant) inhibition of relative tumor growth (Fig 1A and 1B, S3 Fig). This observation presents an important detail that should be taken into consideration in the course of preclinical testing of cancer immunotherapies even if it lacks clinical relevance.

In our hands, four of the five cytokines studied showed anti-tumor efficacy and one of the cytokine candidates, TNF-α, was so potent when given in combination with T-cells that some tumors disappeared completely. This prevented us from analyzing the immune cell content of the treated tumors, but we propose that the overall anti-tumor effect seen in this group was due to the direct anti-tumor effects of TNF-α on one hand [13] and immunological synergy with T-cell therapy on the other. A likely scenario was that TNF-α affected the anti-tumor immune response indirectly via inducing killing of tumor cells and promoting destruction of tumor-associated vasculature [13]. However, we could not investigate this further since advanced necrosis of tumors (day 4 post-transfer) and the cures (day 14 post-transfer) resulted in complete lack of viable tumor material for flow cytometric analysis.

IL-2 is frequently used concomitantly with adoptive T-cell therapies and intratumoral administration of IL-2 has previously been shown to induce infiltration of CD4^+^, CD8^+^ T cells and APCs in preclinical models [37,38]. Our results suggest that in addition to enhanced tumor-infiltration of these immune cells, intratumoral rIL-2 treatment also augments the activation of CD11c^+^ dendritic cells and CD8^+^ TILs (Figs 3B and 5C). This may indicate that tumor-induced tolerance was partly lifted as tumor-induced immunosuppression usually prevents APC-mediated T-cell activation [31]. This conclusion is also supported by the observation that intratumoral administration of IL-2 had the most prominent impact on the downregulation of T-cell anergy markers CTLA-4 and PD-1 (Fig 6A and 6B, Fig A in S4 Fig), both of which contribute strongly to T-cell hypofunction [39]. On the other hand, IL-2 increased overall tumor cytokine secretion with no clear bias toward pro-inflammatory or anti-tumor phenotype (Fig 2A–2H), and IL-2 injections increased the relative amount of Foxp3^+^ CD25^+^ CD4^+^ regulatory T-cells in the tumors (Fig D in S3 Fig), suggesting simultaneous induction of both anti-tumor and immunosuppressive pathways. Specifically, stimulation of anti-tumor CD8+ T-cells, including the OT-I graft and endogenous TILs, is clearly a desirable effect, while untoward effects include the aforementioned stimulation of Tregs. This problem could be overcome by using variants of IL-2 which display reduced stimulation of CD25 while retaining the features sought [40].

Tumor associated macrophages (TAM) and myeloid-derived suppressor cells (MDSC) constitute recently identified immune cell populations within the tumor, with correlation to poor prognosis [41,42]. Reflecting the immunosuppressive nature of these cells, intratumoral administration of GM-CSF and subsequent poor tumor control was accompanied by tumor-infiltration of M2-polarized macrophages and monocytic MDSCs (Figs 1A, 4D and 4F). Since similar effects were not achieved when endogenous GM-CSF was produced by host immune cells (Fig 2E), it is plausible that exogenous GM-CSF given in unregulated supraphysiological concentrations induces immunosuppression [43]. This may implicate that the dose, timing and exposure time of immunomodulatory cytokines on the tumor microenvironment is of importance in context of adoptive T-cell therapy, especially in the case of GM-CSF which has shown promising signs of anti-tumor immune stimulation when used in optimal doses [44]. Our results with recombinant murine GM-CSF also revealed that while the total level of tumor-infiltrating DCs and CD8^+^ T-cells was increased, neither DC maturation nor T-cell activation was enhanced (Figs 3A, 3B, 5A and 5C). GM-CSF has been successfully used to augment anti-tumor immune responses [44,45], but we found that it can induce immune tolerance rather than activation, as reported by Bronte et al [43]. Since GM-CSF employed by several immunotherapeutic approaches such as cancer vaccines and oncolytic viruses [46–48], the aforementioned observations provide important insights into immunobiology of GM-CSF when used as a bolus dose; as opposed to protracted lower-level production locally in a tumor by a viral vector [48].

One factor that might explain the lack of complete eradication of tumors even after combination therapy is the low level of MHC class I molecules expressed on B16 tumor cells, which might prevent tumor cell killing by TILs (Fig 1E). MHC molecules in mice, known as HLA (Human Leukocyte Antigen) in humans, are required for presentation of tumor epitopes to the T-cell receptor [49]. HLA has been reported to be downregulated in several cancer types [50], which results in immune escape through the inability of anti-tumor T-cells to recognize their target. This is of special concern in cell therapies based on TILs or tumor-antigen specific TCR. However, these issues can be circumvented by the use of CAR T-cell therapies [51] which do not rely of MHC/HLA. In our approach, tumor-treatment with interferon-γ resulted in increased expression of MHC class I on tumor cells over non-injected tumors (Fig 1E). Interestingly, we also observed lower levels of activated CD8+ TILs in the IFN-γ-treated mice compared to IFN-α2 mice (Fig 5C), whereas treatment efficacy remained identical in these groups (Fig 1A), suggesting that enhanced tumor cell recognition can compensate for the scarcity of functional T-cells. Finally, IFN-α2 and IL-2 also induced tumor accumulation of NK cells (Fig 4B), which are capable of killing tumor cells expressing low levels of MHC class I [52] and thus compensate for the poor tumor recognition of T-cells. Thus, MHC-I levels are not automatically predictive of therapeutic efficacy even with approaches relying on it for efficacy, when immunostimulatory cytokines are employed. It is, however, possible that qualitative differences in immune responses between different cytokines masked the cytokine-specific role of MHC-I expression in anti-tumor efficacy, i.e. whereas therapeutic efficacy with IFN-γ may depend on MHC-I, efficacy with IFN-α2 and IL-2 might not.

Many established solid tumors are infiltrated by diverse leukocyte subsets including both myeloid- and lymphoid-lineage cells, and the tumor microenvironment plays a major role in delineation of the phenotypic profile and activation status of these cells [53,54]. More importantly, the balance between anti-tumor and pro-tumor immune cells may determine the outcome of cancer immunotherapy [54] and thus encourages closer scrutiny. In our hands, IFN-α2 and IL-2 yielded the best results in terms of T-cell activation versus anergy (Figs 5C and 6A–6B), while IFN-γ treatment decreased expression of exhaustion markers on TILs and increased the number of intratumoral central memory T-cells (Figs 5B and 6A–6B). The majority of tumor-infiltrating myeloid cells identified in these three treatment groups were NK cells and possibly M1 macrophages (Fig 4B–4C), both of which may facilitate T-cell functions [55,56]. Thus it can be argued that local administration of IFN-α2, IFN-γ or IL-2 favorably alters the myeloid-lymphocyte balance and makes the tumor less resistant to immune cell attack.

The terrible pharmacokinetics of recombinant cytokines has necessitated high doses when used systemically, which can be counterproductive as seen for IL-2 for example [20]. An attractive implementation of this approach could include the use of vectors, which can mediate long-term expression, thus solving the problem of short half-life of recombinant cytokines. Moreover, especially viral vectors such as adenoviruses can yield high concentrations locally for protracted periods, which low concomitant concentrations systemically [57]. A further improvement of this approach would be the use of adenoviral vectors that are replication-competent only in tumor cells; one of the first phases of replication of the virus is replication of the genome, resulting in 10000-fold amplification of the transgene expression cassette. If the virus is designed in a way linking transgene expression to virus replication, viral expression of immunomodulatory cytokines *in situ* could potentially offer a safer and more tumor-selective option than recombinant cytokines, as transgene expression would not occur in non-transcomplementing (= non-tumor) cells [57].

In summary, incorporation of immunomodulatory cytokines IFN-α2, IFN-γ, TNF-α and IL-2 into treatment regimen can alter the tumor microenvironment in favor of T-cell function, whereas in situ injections of GM-CSF can induce and sustain highly immunosuppressive immune cell populations within the tumor, thus leading to poor tumor growth control. These results have important implications in several experimental immunotherapies, and provide a strong rationale for adaptation of direct or vectored cytokine administration into T-cell therapy regimens.

## Supporting Information

S1 Fig Treatment schedule. Female C57BL/6 mice were implanted with 2,5x10^5^ B16.OVA cells subcutaneously into the right flank (1 tumor/mouse). 10 days post-implantation mice were divided into groups and injected intraperitoneally with 2x10^6^ polyclonally activated CD8a+ enriched OT-I lymphocytes. Beginning on the same day, tumors were injected with PBS or with one of the recombinant murine cytokines diluted in PBS. One control group of mice received only adoptive transfer of OT-I cells and the tumors were left non-injected to avoid immune responses generated by physical (needle) manipulation of the tumor microenvironment. Intratumoral injections were continued for 5 consecutive days per week. A set of mice were sacrificed (SAC) and organs were harvested for analysis on days 4 and 14 post-transfer.
(TIF)

S2 Fig Intratumoral administration of TNF-α combined with adoptive transfer of OT-I cells results in anti-tumor efficacy. Mice bearing B16.OVA flank tumors were adoptively transferred with 2x10^6^ CD8a^+^ enriched OT-I lymphocytes intraperitoneally and tumors were either not injected or injected with PBS or recombinant cytokines in PBS (n = 10). Tumor growth was monitored every 2–3 days with an electronic caliper. (Fig A) Absolute tumor volumes (mm^3^) of all groups and (Fig B) relative tumor volumes (% of day 0 volume) of TNF-α treatment group. Data presented as mean ± SEM. ****P≤ 0.0001 by repeated measures ANOVA.
(TIF)

S3 Fig Lymphocyte subsets in the tumors following cytokine treatment. Mice with B16.OVA flank tumors were treated with adoptive transfer of 2x10^6^ CD8a^+^ enriched OT-I lymphocytes intraperitoneally and with 50 μl PBS or recombinant cytokine in PBS intratumorally (n = 5). Levels of tumor-infiltrating (Fig A) CD45^+^ leukocytes, (Fig B) CD3^+^ T-lymphocytes, (Fig C) CD4^+^ T-lymphocytes and (Fig D) proportion of regulatory T-cells of CD4^+^ T-cells were assessed by flow cytometry on day 14 post-transfer. (Figs E–F) Amounts of endogenous CD8+ TILs targeting melanoma-associated antigens TRP-2 and gp100 were quantified on day 14 post-transfer by pentamer staining and flow cytometry. Data presented as mean ± SEM. *P ≤ 0.05, **P≤ 0.01 by one-way ANOVA followed by Tukey’s post-hoc test.
(TIF)

S4 Fig Expression of anergy markers on CD8+ TILs on day 4 post-transfer. B16.OVA-bearing mice were injected with 2x10^6^ CD8a^+^ enriched OT-I lymphocytes intraperitoneally and beginning on the same day, tumors were injected with either PBS or recombinant cytokine in PBS or left non-injected (n = 5). Proportion of CD3^+^ CD8^+^ TILs expressing surface anergy markers (Fig A) CTLA-4 and (Fig B) PD-1 was analyzed by flow cytometry on day 4 post-transfer. Data presented as mean ± SEM. *P ≤ 0.05, **P≤ 0.01 and ***P≤ 0.001 by one-way ANOVA followed by Tukey’s post-hoc test.
(TIF)

S5 Fig Heat map summarizing the differenct aspects of immunostimulatory cytokines in the modulation of tumor microenvironment. Decrease (red), increase (green) or no change (gray) in activation status or proportion of different cell populations following cytokine treatment compared to non-injected tumors.
(TIF)
