# Supplementary figures and images for: Favorable Alteration of Tumor Microenvironment by Immunomodulatory Cytokines for Efficient T-Cell Therapy in Solid Tumors
Source: PLoS One. 2015 Jun 24;10(6):e0131242. doi: 10.1371/journal.pone.0131242 (PMC4479879; doi:10.1371/journal.pone.0131242)

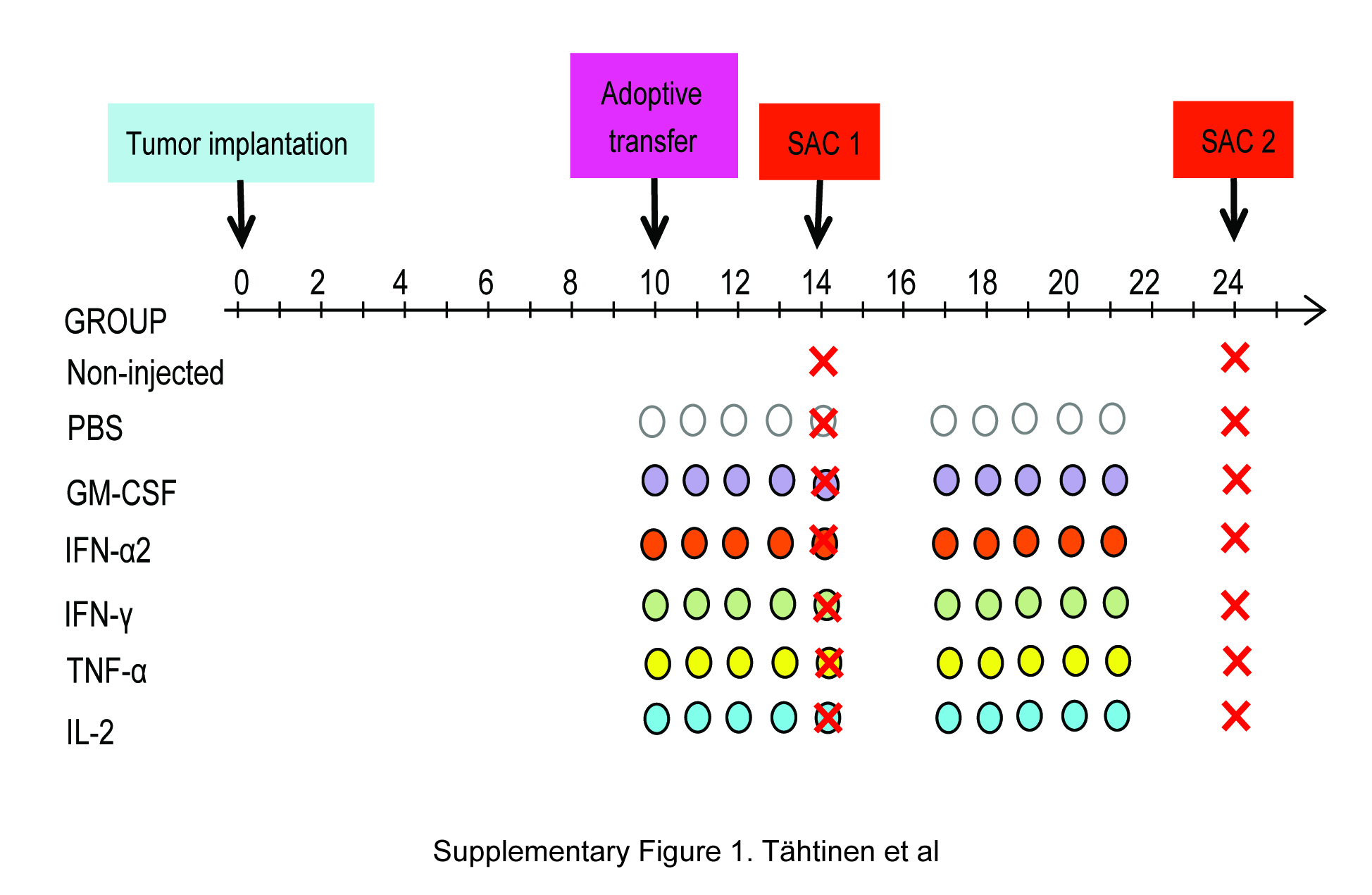

Supplement: S1 Fig — Female C57BL/6 mice were implanted with 2,5x10^5^ B16.OVA cells subcutaneously into the right flank (1 tumor/mouse). 10 days post-implantation mice were divided into groups and injected intraperitoneally with 2x10^6^ polyclonally activated CD8a+ enriched OT-I lymphocytes. Beginning on the same day, tumors were injected with PBS or with one of the recombinant murine cytokines diluted in PBS. One control group of mice received only adoptive transfer of OT-I cells and the tumors were left non-injected to avoid immune responses generated by physical (needle) manipulation of the tumor microenvironment. Intratumoral injections were continued for 5 consecutive days per week. A set of mice were sacrificed (SAC) and organs were harvested for analysis on days 4 and 14 post-transfer. (TIF) [file pone.0131242.s001.tif]

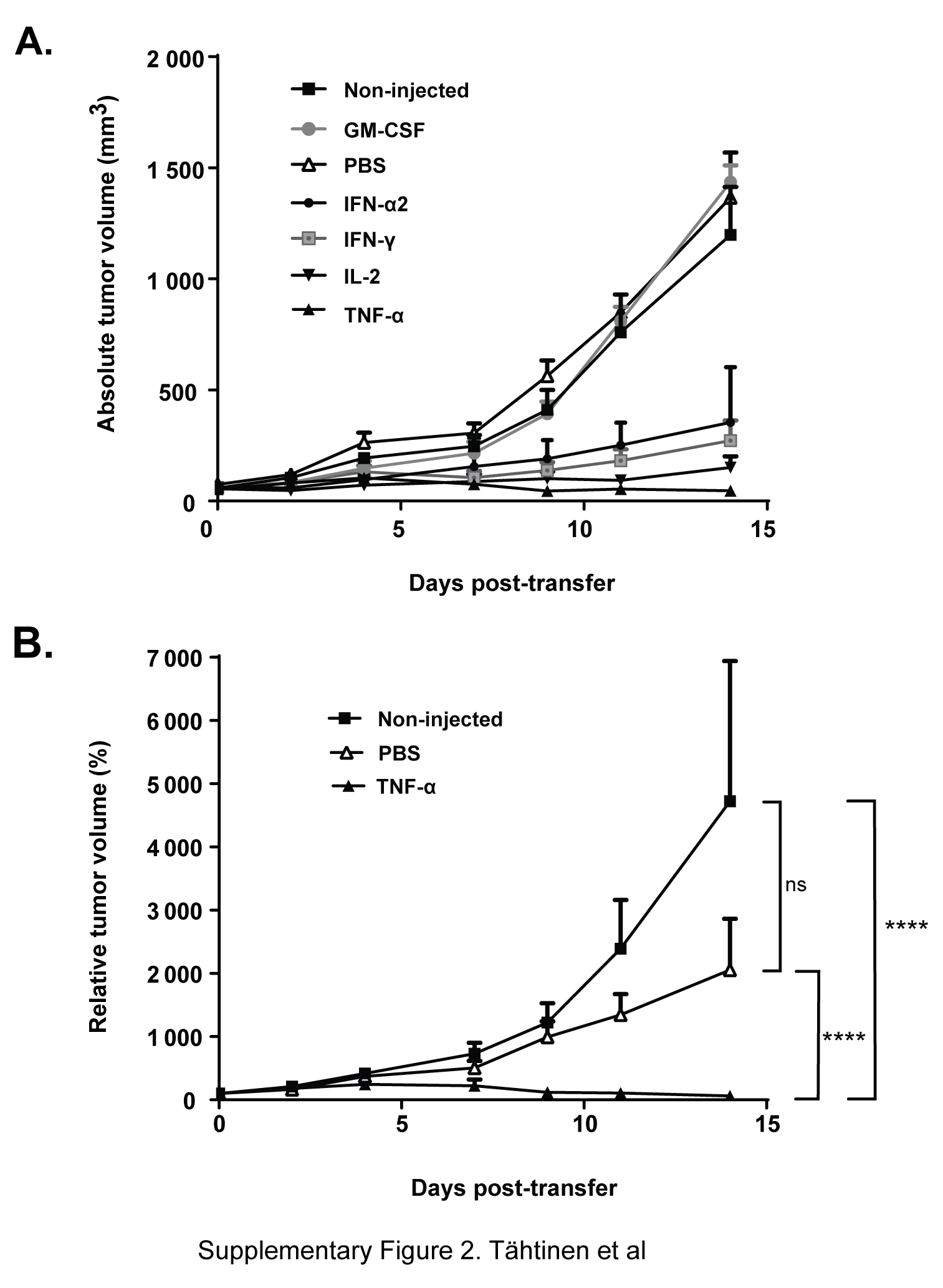

Supplement: S2 Fig — Mice bearing B16.OVA flank tumors were adoptively transferred with 2x10^6^ CD8a+ enriched OT-I lymphocytes intraperitoneally and tumors were either not injected or injected with PBS or recombinant cytokines in PBS (n = 10). Tumor growth was monitored every 2–3 days with an electronic caliper. (Fig A) Absolute tumor volumes (mm3) of all groups and (Fig B) relative tumor volumes (% of day 0 volume) of TNF-α treatment group. Data presented as mean ± SEM. ****P≤ 0.0001 by repeated measures ANOVA. (TIF) [file pone.0131242.s002.tif]

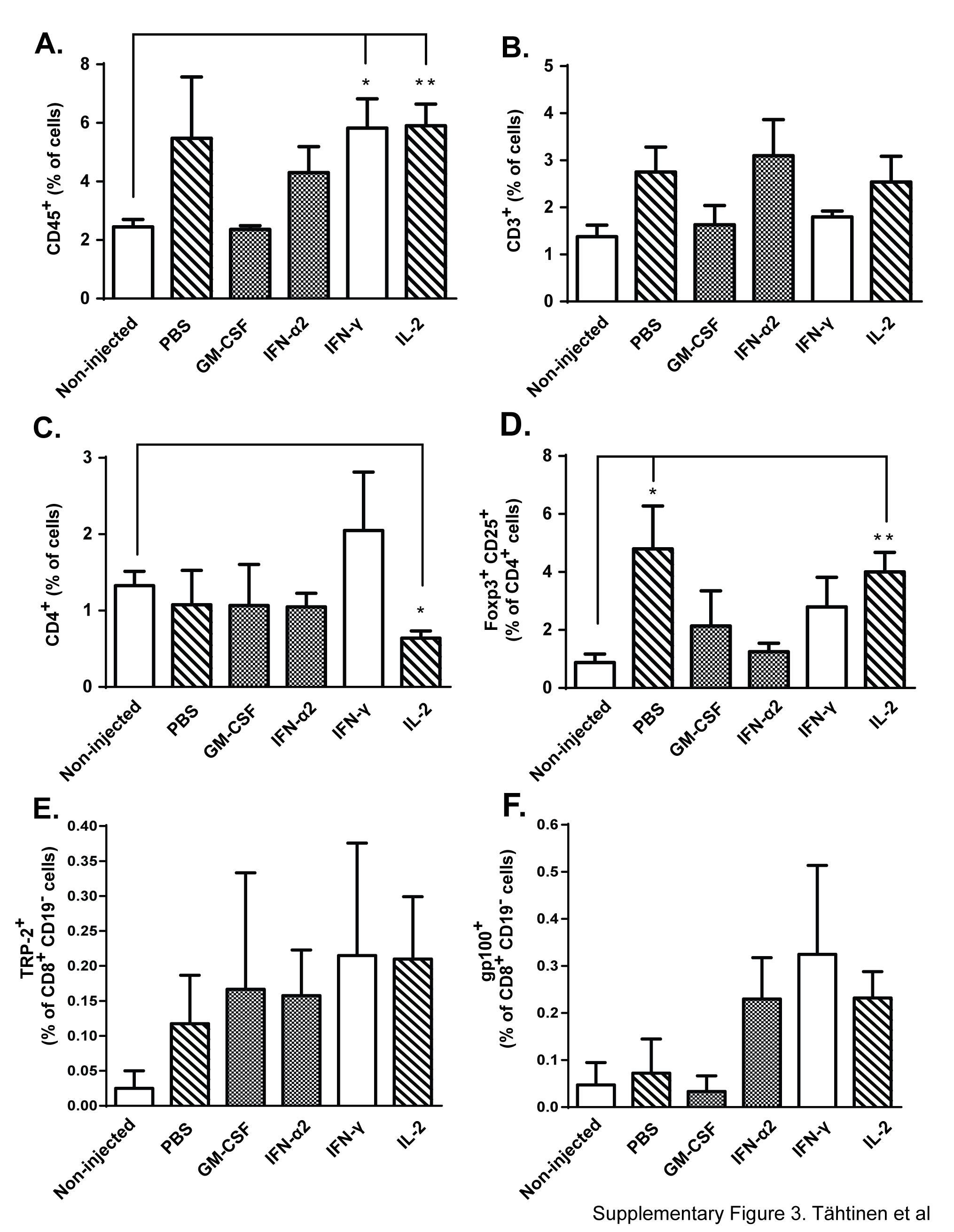

Supplement: S3 Fig — Mice with B16.OVA flank tumors were treated with adoptive transfer of 2x10^6^ CD8a+ enriched OT-I lymphocytes intraperitoneally and with 50 μl PBS or recombinant cytokine in PBS intratumorally (n = 5). Levels of tumor-infiltrating (Fig A) CD45+ leukocytes, (Fig B) CD3+ T-lymphocytes, (Fig C) CD4+ T-lymphocytes and (Fig D) proportion of regulatory T-cells of CD4+ T-cells were assessed by flow cytometry on day 14 post-transfer. (Figs E–F) Amounts of endogenous CD8+ TILs targeting melanoma-associated antigens TRP-2 and gp100 were quantified on day 14 post-transfer by pentamer staining and flow cytometry. Data presented as mean ± SEM. *P ≤ 0.05, **P≤ 0.01 by one-way ANOVA followed by Tukey’s post-hoc test. (TIF) [file pone.0131242.s003.tif]

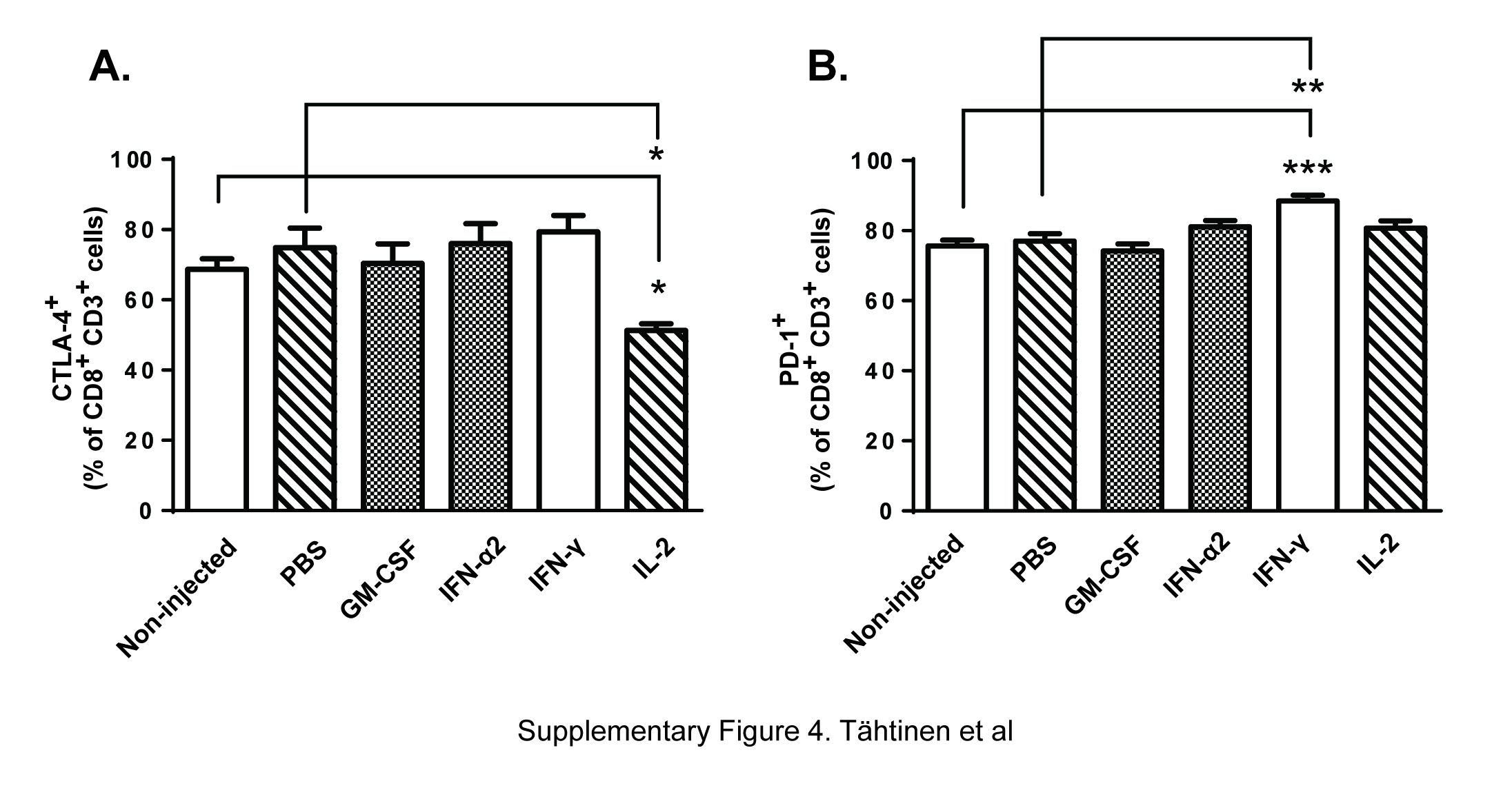

Supplement: S4 Fig — B16.OVA-bearing mice were injected with 2x10^6^ CD8a+ enriched OT-I lymphocytes intraperitoneally and beginning on the same day, tumors were injected with either PBS or recombinant cytokine in PBS or left non-injected (n = 5). Proportion of CD3+ CD8+ TILs expressing surface anergy markers (Fig A) CTLA-4 and (Fig B) PD-1 was analyzed by flow cytometry on day 4 post-transfer. Data presented as mean ± SEM. *P ≤ 0.05, **P≤ 0.01 and ***P≤ 0.001 by one-way ANOVA followed by Tukey’s post-hoc test. (TIF) [file pone.0131242.s004.tif]

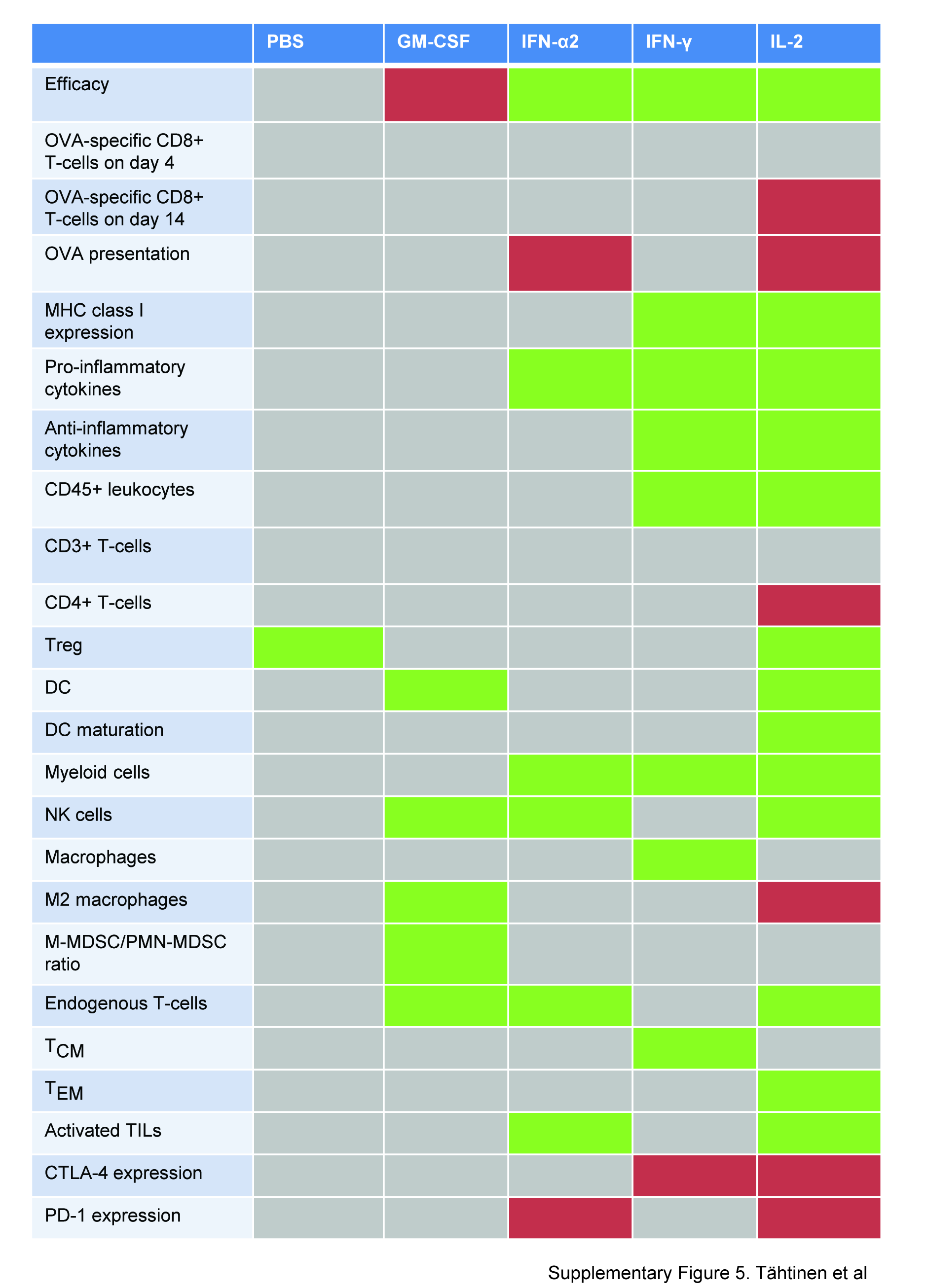

Supplement: S5 Fig — Decrease (red), increase (green) or no change (gray) in activation status or proportion of different cell populations following cytokine treatment compared to non-injected tumors. (TIF) [file pone.0131242.s005.tif]
